# Association of Polymorphisms in STRA6 and RARRES2 Genes with Type 2 Diabetes in Southern Han Chinese

**DOI:** 10.1155/2016/6589793

**Published:** 2016-07-03

**Authors:** Han-Wei Huang, Bi-Yu Liang, Yun-Xi Li

**Affiliations:** ^1^Department of Endocrinology, The Affiliated Zhongshan Hospital of Guangdong Medical College, Zhuyuan Road 18, Xiaolan, Zhongshan 528415, China; ^2^Department of Clinical Laboratory, The Affiliated Zhongshan Hospital of Guangdong Medical College, Zhuyuan Road 18, Xiaolan, Zhongshan 528415, China

## Abstract

Stimulated by retinoic acid gene homolog 6 (STRA6) and retinoic acid receptor responder 2 (RARRES2) are candidate genes involved in the pathogenesis of type 2 diabetes mellitus (T2DM). Three tag-SNPs in STRA6 and one in RARRES2 gene were selected and genotyped with TaqMan or PCR-RFLP method in 603 populations (571 patients with T2D versus 632 control subjects) in Southern Han Chinese. We estimated the interactions between T2DM risk and genetic variants in the STRA6 and RARRES2 genes using polymerase chain reaction. Rs736118 in STRA6 gene were significantly associated with T2DM occurrence in the recessive genetic model. The genotype of rs974456 was significantly associated with T2DM in the dominant genetic model correlated to sex, MBI, and triglyceride. However, the association of other SNPs with T2DM was not found. Furthermore, smoking history and other factors may be independent risk factors for the incidence of T2DM. This study suggested that a role of STRA6 polymorphism could also be of value in predicting the risk of T2DM while RARRES2 polymorphism could not predict the risk of T2DM.

## 1. Introduction

Type 2 diabetes mellitus (T2DM) is a kind of metabolic syndrome primarily characterized by disturbance of carbohydrate metabolism, which is caused by relative insulin deficiency and/or insulin resistance. Meanwhile, T2DM is an illness for polygenic inheritance resulting from genetic factor and environmental factor [1]. Present studies focused on the related candidate genes of the insulin signal transduction pathway and lipid metabolism pathway [2]. The effect and incidence of candidate genes are different. There may also be interactions among different genes which can bring about changes in protein expression, lead to abnormal signal transduction pathways or metabolic pathways, result in glucolipid metabolism disorder, and cause T2DM. Furthermore, the effect of gene-environment interaction can lead to different risk of T2DM that different individual suffers from. At present, as an endocrine organ, adipose tissue gets more attention in etiology research of T2DM. Various adipokines secreted by adipose tissue are regarded as endogenous signal molecules involving glucolipid metabolism, disturb insulin signal pathways [3], and promote the development of T2DM.

Retinol binding protein 4 (RBP4) is an adipokine; its serum level is closely related to insulin resistance and T2DM [4–6]. Moreover, studies in different area and different population showed that RBP4 gene polymorphism was significantly associated with T2DM [7–9]. As a cell surface receptor, stimulated by retinoic acid gene homolog 6 (STRA6) is also a kind of cytokine receptor activating JAK2-STAT5 signal pathway and inducing the production of SOCS-3 by combining with retinol-RBP4 complexes [10]. But the SOCS-3 may suppress JAK-STAT pathway through negative feedback which interferes with insulin signal transduction [11] and causes T2DM. STRA6 gene locates on chromosome 15q24.1 region and contains 20 exons and 19 introns, which encodes a protein of 667 amino acids. Nair et al. have found an association between STRA6 rs974456, rs736118, and rs4886578 polymorphisms and T2DM in a South Indian population [12]. They have found that the haplotype (TAA) may reduce the risk of T2DM, which was considered as a protective factor for T2DM. Nevertheless, Juanjuan has reported that STRA6 rs974456 polymorphism may increase the susceptivity of T2DM and that the minor allele (T allele) and genotype (TT) were independently risk factors for T2DM in Han population in Tianjin, China [13].

Retinoic acid receptor responder 2 (RARRES2), which was known as chemerin or tazarotene-induced gene 2, was found to be highly expressed in adipose tissue. It has been found that serum RARRES2 level was significantly associated with T2DM [14]. Moreover, it can be effective in reducing the serum RARRES2 level that patients in T2DM get exercises and feed on low-calorie diet, which can contribute to improve insulin sensitivity [15]. Another study has showed that serum RARRES2 level was correlated with metabolic syndrome indicators positively including body mass index, waist-hip ratio, and blood pressure [16]. Elevated serum RARRES2 level may lead to insulin resistance in primary human skeletal muscle cells through activating p38 mitogen-activated protein kinase, nuclear factor-kappa B and extracellular signal-regulated kinase (ERK1/2) [17]. Besides, RARRES2 may reduce insulin-stimulated insulin receptor substrate-1 tyrosine phosphorylation in 3T3-L1 adipocytes [18] and decrease the glucose transport mediated by glucose transporter-2 in liver tissue [19].

RARRES2 gene is located on chromosome 7q36.1 and is comprised of 6 exons and 5 introns, which encodes a protein of 163 amino acids. Hashemi et al. have found that RARRES2 rs17173608 polymorphisms increased the risk of metabolic syndrome in Zahedan, Southeast Iran, and that the minor allele of RARRES2 rs17173608 (G allele) was a risk factor of metabolic syndrome [20]. Müssig et al. have reported a significantly association between RARRES2 rs17173608 and rs10278590 polymorphisms and visceral adipose tissue in nonobese but not obese individuals [21]. Their findings suggested that common genetic variation in the RARRES2 gene may specifically contribute to the development of visceral adiposity. Furthermore, Min et al. have found the potential association between RARRES2 rs10282458 polymorphism and body mass index in the experimental identification of metabolic syndrome-related genes [22]. These studies all have prompted that RARRES2 gene promotes the occurrence of T2DM by means of disturbing glucose and lipid metabolism signal pathway [23]. Therefore, RARRES2 is thought to be a susceptibility gene for T2DM. We infer that RARRES2 gene polymorphism may be associated with T2DM.

To the best of our knowledge, most studies confined to single gene or locus and there is a large distributional difference in different region or race. The reason for this difference may be that patients with T2DM have different genetic background and that the clinical manifestations caused by risk variants are possibly affected by environmental factors including lifestyle and dietary habit. The geographical environment in Southern China provides abundant diet resources, which makes the people of Southern China pay special attention to “eat.” Moreover, people of Han population in Southern China prefer to have porridge which is rich in carbohydrates and has high glycemic index. They develop their habits of “morning tea,” “afternoon tea,” and “night snack” gradually. It is easy to exacerbate the burden of pancreas islet and glycolipid metabolic disorders for frequent eating. These may be the major reasons for T2DM, metabolism syndrome, and hyperlipidemia in Southern China. It is possible for humans to suffer from T2DM under the influence of environmental factors. Therefore, the present study was aimed to find out the possible association between STRA6 and RARRES2 gene polymorphisms and T2DM in Han population of Southern China. Furthermore, we performed the interaction association analysis on gene-gene and gene-environment factors to evaluate the risk of T2DM and provide the theoretical basis for revealing the genetic susceptibility of T2DM.

## 2. Materials and Methods

### 2.1. Subjects

This study adopted a case-control design, including 571 patients with T2DM from endocrine department of nine affiliated hospitals in Zhanjiang, Maoming, Dongguan, Shenzhen, and Zhongshan in Southern China and 632 normal control subjects with physical examination during the same period and in the same hospitals. Individuals of T2DM were recruited according to 1999 WHO recognized standards. We excluded type 1 diabetes mellitus, gestational diabetes, and other special type diabetes. Control subjects did not carry family history of hereditary diseases. We excluded people who suffered from acute stress condition (such as acute myocardial infarction, cerebral infarction, infection, trauma, and surgery) and liver and kidney dysfunction. The fasting plasma glucose (FBG) in control subjects was normal (FBG < 6.1 mmol/L).

### 2.2. Date and Blood Samples Collection

Investigators in the uniform training did the standardized questionnaires of study subjects and their height, weight, and blood pressure were measured. The peripheral blood of subjects with an empty belly at least for 12 hours was collected in the early morning and was used for detection of blood glucose, lipids, and other clinical biochemical indicators which were recorded by the investigators. Moreover, the genomic DNA was extracted from peripheral blood. The study was approved by the Ethics Committee of Guangdong Medical College. All subjects provided written informed consent.

### 2.3. Detection of Gene Polymorphisms

The peripheral blood genomic DNA was extracted by Tiangen genomic DNA extraction kit (Beijing, China) and the concentration and purity of genomic DNA were detected by ND-1000 nucleic acid protein content detector (Nanodrop Spectrophotometer). The DNA was diluted to the final concentration of 20–40 ng/*μ*L before PCR reaction. The following was PCR reaction system (5 *μ*L): genotyping assay (20x), 0.25 *μ*L; Universal PCR Master Mix (2x), 2.5 *μ*L; genomic DNA, 0.5 *μ*L; and ddH_2_0, 1.75 *μ*L. Cycling parameters were as follows: initial denaturation of one cycle of 10 min at 95°C, followed by 40 cycles of 15 s at 95°C and 60 s at 60°C and followed by melting curve analysis. We set up two wells with ddH_2_O instead of genomic DNA as the negative control in each of 384-well plates during the experiment. We put the 384-well plates for PCR amplification in ABI 7900 HT fluorescence quantitative PCR instrument and read the fluorescence signal after PCR reaction. We determined genotype as wild homozygous, homozygous, or heterozygotes risk variants finally with SDS 2.0 image analysis software program “Allelic Discrimination” that relied on the FAM and VIC fluorescence intensity of each alleles. Assay ID of TapMan probe was shown in Table 1 (ABI, USA).

### 2.4. Statistical Methods

Comparisons of all variables between T2DM and control subjects were carried out by chi-square test for nominal variables or *t*-test for continuous variables. Nominal variables were expressed in percentage (%) and continuous variables were expressed in the form of means ± standard deviations. The analysis was performed by SPSS (version 16.0). Genotype distributions for all studied SNPs were tested for Hardy-Weinberg equilibrium (HWE) by chi-square tests and no significant deviation was found in control subjects. We tested the association of the single SNP polymorphisms with type 2 diabetes using Pearson chi-square test and logistic regression. Chi-square test was used for frequencies of haplotypes analysis. Logistic regression was also used for SNP-SNP interactions analysis and gene-environmental interaction studies. These were performed by PLINK software. Difference was considered to be statistically significant with *P* < 0.05.

## 3. Results

### 3.1. Clinical Characteristics of the Enrolled Subjects

There were 1203 subjects in our case-control study including 571 T2DM patients (288 males and 283 females) and 632 healthy people (399 males and 233 females). The age, BMI, fast blood glucose, triglyceride, and low density lipoprotein-cholesterol were significantly different in diabetes and control groups (*P* < 0.05). BMI, serum level of fast blood glucose, and triglyceride were significantly higher in T2DM subjects than in healthy subjects. However, the age, hypertension, serum level of total cholesterol, and high density lipoprotein-cholesterol had no difference between the T2DM and control groups (*P* > 0.05). Clinical characteristics of the enrolled subjects were presented in Table 2.

### 3.2. Analysis of Correlation between Alleles and T2DM

Minor allele T frequencies of SNP rs736118 of STRA6 were 30.74% (351/791), which was not statistically significant between T2DM group and control group when multiple-test adjusted values (FDR) were used (*P* > 0.05). Allele frequencies distribution of SNP rs4886578 and rs974456 of STRA6 and SNP rs17173617 of RARRES2 was not statistically significant between T2DM group and control group (*P* > 0.05). Analysis of correlation between alleles and T2DM was presented in Table 3.

### 3.3. Association of Genotype between Controls and T2DM

SNPs of rs736118, rs4886578, and rs974456 in STRA6 were all C/T dimorphism. Their wild alleles are C, and risk variants alleles are T. Their genotypes were wild homozygous CC, heterozygous risk variants CT, and homozygous risk variants TT. The distribution of genotype frequencies of SNP rs736118 and rs974456 was significantly different in T2DM and control group when FDR was used (rs736118: *P* = 0.046; rs974456: *P* = 0.040). The distribution of genotype frequencies of SNP rs4886578 was not significantly different in T2DM and control group (*P* > 0.05). SNPs (rs17173617) in the RARRES2 were all G/C dimorphism. Their wild alleles are G, and risk variants alleles are C. Their genotypes were wild homozygous GG, heterozygous risk variants GC, and homozygous risk variants CC. The distribution of genotype frequencies of rs17173617 was not significantly different in T2DM and control group (*P* > 0.05). Association of genotype between controls and T2DM was presented in Table 4.

### 3.4. Correlation Analysis in Three Genetic Models

Allele gene T of SNP rs736118 of STRA6 was significantly associated with T2DM under recessive genetic model when FDR was used (OR = 0.60, 95% CI: 0.42–0.87, *P* = 0.028), but not under the dominant genetic model and the additive genetic model (*P* > 0.05). It showed that homozygous TT protected Southern Han Chinese from T2DM. Allele gene T of SNP rs974456 of* STRA6* gene was significantly associated with T2DM under the dominant genetic model when FDR was used (OR = 0.73, 95% CI: 0.57–0.94, *P* = 0.048), but not under the additive genetic model and recessive genetic model, which suggested that people that had allele gene T could significantly reduce the risk of T2DM (*P* > 0.05). No association between T2DM and rs4886578 or rs17173617 was found in allelic and genotypic association analysis under three models (*P* > 0.05). Correlation Analysis in three genetic models was presented in Table 5.

After the adjustment of sex, BMI, and triglyceride, both SNP rs736118, rs4886578, and rs974456 of* STRA6* gene and SNP rs17173617 of* RARRES2* gene had no association under the three genetic models in genotypic association analysis when FDR was used (*P* > 0.05). Correlation Analysis after the adjustment in three genetic models was presented in Table 6.

### 3.5. Analysis of Interactions between SNP and Smoking History

There is no statistical significance in relationship between smoking history and effect of STRA6 on T2DM under the additive model, dominant model, and recessive model (*P* > 0.05). Correlation Analysis in three genetic models was presented in Table 7.

## 4. Discussion

In the present study, we selected STRA6, RARRES2 gene related T2DM as candidate genes, screened SNP rs736118, rs4886578, and rs974456 of STRA6 gene, and SNP rs17173617 of RARRES2 gene as candidate gene polymorphisms; we found that genotype TT of SNP rs736118 on STRA6 was significantly associated with T2DM and protected human against T2DM, also after the adjustment of sex, BMI, and triglyceride. Moreover, it had been firstly found that C/T dimorphism of SNP rs736118 on STRA6 was significantly associated with T2DM in our study, which has not also been reported even in other ethnic groups, but Nair et al. [12] showed that G/A on its complementary strand was associated with T2DM. As a cell surface receptor of RBP4, STRA6 activated JAK2-STAT5 signal pathway, induced production of SOCS-3 by combining with retinol-RBP4 complexes, and led to insulin resistance to promote the occurrence of T2DM. For SNP of rs736118, C→T conversion will cause amino acid change of STRA6 from methionine to tyrosine. In particular, the location of this SNP is in the c-terminal of STRA6, which has been implicated in signal transduction via phosphorylation. Tyrosine conversion could potentially have an impact on the signal transduction pathway. For SNP of rs974456 is within an intron of STRA6, therefore, it is unlikely to cause a biochemical change in STRA6. Genotype TT of SNP rs974456 on STRA6 was significantly associated with T2DM; moreover, the carrier with allele T (TT + TC) has significantly lower risk to T2DM than carrier with genotype CC alone after logistic regression analysis. There was still a protective effect, but the difference was not statistically significant after the adjustment of sex, BMI, and triglyceride. SNP rs974456 and rs4886578 of STRA6 may be not associated with incidence of T2DM in Southern Han Chinese. However, Nair et al. [12] found that SNP rs974456 and rs4886578 were associated with T2DM in the South Indian population. Juanjuan [13] has reported that genotype TT on SNP rs974456 may increase susceptibility to T2DM, which was an independent risk factor of T2DM. The explanation may be that frequencies distributions of genetic polymorphisms in the population are different in different races and areas, which have a strong effect on the filtration of T2DM pathogenesis-related gene. Moreover, interaction between genes is different from difference of genetic structure on different ethnic origin, which may be affected by diet and lifestyle and other environmental factors [24, 25].

As to the mechanisms linking SNP rs736118 of STRA6 and T2DM, a plausible interpretation of the experimental data is that C/T dimorphism on SNP rs736118 of STRA6 was in exon coding regions, which resulted in changes of protein function and affected the development of T2DM. It had been found in other studies that G/A dimorphism on SNP rs736118 resulted in changes of DNA base sequence, which caused amino acid change from methionine to isoleucine and reduced the risk of T2DM [12]. Moreover, supplement of isoleucine can stimulate insulin secretion and improve glucose metabolism [26]. However, it has not been reported whether C→T conversion will cause amino acid change from methionine to tyrosine.

As to the relation between SNP rs736118 and rs974456 of STRA6 and T2DM, we found that frequencies distributions of genotype of SNP rs736118 and rs974456 were statistically significant between cases and controls. But after the adjustment of sex, BMI, and triglyceride, the difference was not statistically significant when FDR was used, suggesting that sex, BMI, and triglyceride may increase the risk of T2DM in people with risk variants in* STRA6* gene. Our study found that triglyceride levels and BMI were significantly higher in case group than in control group, which may affect association analysis between gene polymorphisms and T2DM. Previous studies have found that levels of serum RARRES2 were closely associated with T2DM and its chronic cardiovascular and cerebrovascular complications. SNP rs17173608 of RARRES2 can increase the risk of suffering from metabolic syndrome and SNP rs10278590 and rs 10282458 can promote formation of visceral fat [20–22] to increase the risk of T2DM. However, it was not found that RARRES2 polymorphism was directly related to T2DM.

On the other hand, we study eight haplotypes among SNP rs736118, rs4886578, and rs974456 of STRA6, but none of the haplotypes was associated with T2DM. At the same time, we found no interaction between STRA6 and RARRES2. There was no interaction among SNPs that were not associated with T2DM.

Furthermore, because there is a clear relationship between incidence of T2DM and smoking history, drinking history, hypertension history, or T2DM family history [27–32], we investigated whether the interaction between STRA6, RARRES2 candidate genes and environmental factors would impact the occurrence of T2DM. In our study, the results show that there were no relationships between effect of STRA6 on T2DM and smoking history, drinking history, overweight, overweight, hypertension history, serum total cholesterol levels, diet, and exercise and other factors may be independent risk factors for the incidence of T2DM in Southern Han Chinese.

In summary, our study found SNP rs736118 of STRA6 was associated with type 2 diabetes mellitus and protected human against T2DM, but the interaction between STRA6 gene and drinking history, T2DM family history, and overweight easily leads to the occurrence of T2DM.

However, limitations should be noted in the present study. Firstly, these results are only preliminary analysis of genetic susceptibility to T2DM, which is to understand the roles of candidate genes in the development of type 2 diabetes. Secondly, further functional analysis is needed to confirm these results. Thirdly, we need to replicate our finding in additional samples in future studies.

## Figures and Tables

**Table 1:** Probes of all SNPs.

Article number	SNP	Sequence
C_966873_10	rs736118	CAGGTGGCCAC[C/T]ATGGCACCCAC
C_32357728_10	rs4886578	ATTTTCCTGGCG[C/T]TCACTCTGTGTG
C_3152257_20	rs974456	ACTTCCCCTTCT[C/T]GAGCCCTGCAGA
C_33580177_10	rs17173617	ATCTTGCCCATT[C/G]TGTCTCTCCCTGG

**Table 2:** Clinical characteristics of the enrolled subjects.

Index	Cases	Controls	*P* value
Number	571	632	—
Male (%)	50.4	63.1	<0.0001
Hypertension (%)	41.5	41.4	0.973
Age (years)^*∗*^	58.29 ± 10.52	58.26 ± 10.40	0.967
BMI (kg/m^2^)^*∗*^	24.51 ± 3.81	23.39 ± 3.31	<0.0001
FBG (mmol/L)^*∗*^	10.79 ± 4.73	5.41 ± 0.99	<0.0001
TC (mmol/L)^*∗*^	5.40 ± 1.59	5.43 ± 1.07	0.116
TG (mmol/L)^*∗*^	2.27 ± 2.71	1.36 ± 1.03	<0.0001
HDL-C (mmol/L)^*∗*^	1.42 ± 0.74	1.37 ± 0.38	0.761
LDL-C (mmol/L)^*∗*^	2.89 ± 1.11	3.06 ± 0.69	<0.0001

Note: ^*∗*^values were continuous variables, as mean ± standard deviation given in the form.

**Table 3:** Analysis of correlation between alleles and T2DM.

SNP	Minor allele	MAF of cases	MAF of controls	Major allele	*x*	*P* value	*P* value (FDR)
rs736118	T	351 (0.307)	442 (0.350)	C	4.865	0.027	0.108
rs4886578	T	100 (0.088)	137 (0.108)	C	2.929	0.087	0.174
rs974456	T	490 (0.429)	574 (0.454)	C	1.525	0.217	0.217
rs17173617	C	301 (0.264)	371 (0.294)	G	2.672	0.102	0.136

**Table 4:** Association of genotype between controls and T2DM.

SNP	Genotype	Case		Control		*x*	*P* value	*P* value (FDR)
		Number	%	Number	%			
rs736118	TT	50	8.8	87	13.8	7.565	0.023	0.046
	CT	251	44	268	42.4			
	CC	270	47.3	277	43.8			

rs4886578	TT	6	1.1	7	1.1	3.437	0.179	0.239
	CT	88	15.4	123	19.5			
	CC	477	83.5	502	79.4			

rs974456	TT	121	21.2	123	19.5	9.210	0.010	0.040
	CT	248	43.4	328	51.9			
	CC	202	35.4	181	28.6			

rs17173617	CC	47	8.2	58	9.2	3.060	0.216	0.216
	GC	207	36.3	255	40.3			
	GG	317	55.5	319	50.5			

**Table 5:** Correlation analysis in three genetic models.

SNP	Additive model			Dominance model			Recessive model		
	*P*	*P* (FDR)	OR (95% CI)	*P*	*P* (FDR)	OR (95% CI)	*P*	*P* (FDR)	OR (95% CI)
rs736118	0.030	0.120	0.83 (0.70–0.98)	0.229	0.229	0.87 (0.69–1.09)	0.007	0.028	0.60 (0.42–0.87)
rs4886578	0.090	0.180	0.79 (0.60–1.04)	0.068	0.136	0.76 (0.57–1.02)	0.924	0.924	0.95 (0.32–2.84)
rs974456	0.224	0.224	0.91 (0.77–1.06)	0.012	0.048	0.73 (0.57–0.94)	0.457	0.914	1.11 (0.84–1.47)
rs17173617	0.110	0.147	0.87 (0.73–1.03)	0.080	0.107	0.82 (0.65–1.03)	0.562	0.749	0.89 (0.59–1.33)

**Table 6:** Correlation analysis after the adjustment in three genetic models.

SNP	Additive model			Dominance model			Recessive model		
	*P*	*P* (FDR)	OR (95% CI)	*P*	*P* (FDR)	OR (95% CI)	*P*	*P* (FDR)	OR (95% CI)
rs736118	0.033	0.132	0.81 (0.66–0.98)	0.146	0.195	0.82 (0.63–1.07)	0.026	0.104	0.61 (0.39–0.94)
rs4886578	0.192	0.256	0.82 (0.60–1.11)	0.163	0.163	0.79 (0.57–1.10)	0.884	0.884	0.92 (0.28–2.96)
rs974456	0.554	0.554	0.95 (0.79–1.14)	0.069	0.138	0.77 (0.58–1.02)	0.315	0.630	1.18 (0.86–1.61)
rs17173617	0.068	0.136	0.83 (0.68–1.01)	0.026	0.104	0.74 (0.57–0.97)	0.793	0.835	0.94 (0.59–1.50)

**Table 7:** Analysis of interactions between SNP and smoking history.

SNP	Additive model			Dominance model			Recessive model		
	*P*	*P* (FDR)	OR (95% CI)	*P*	*P* (FDR)	OR (95% CI)	*P*	*P* (FDR)	OR (95% CI)
rs736118	0.543	0.572	1.14 (0.75–1.73)	0.646	0.861	1.14 (0.64–2.03)	0.565	0.595	1.30 (0.54–3.13)
rs4886578	0.671	0.671	0.85 (0.40–1.81)	0.813	0.813	0.91 (0.41–2.00)	0.999	0.999	—
rs974456	0.587	0.783	0.89 (0.58–1.36)	0.057	0.228	0.55 (0.30–1.02)	0.168	0.672	1.62 (0.82–3.21)
rs17173617	0.333	0.351	0.80 (0.51–1.26)	0.297	0.594	0.73 (0.41–1.31)	0.715	0.953	0.82 (0.29–2.35)
